# Peripheral neuropathy secondary to a ‘domino’ liver transplant: a case report

**DOI:** 10.1186/s13256-023-04001-0

**Published:** 2023-06-29

**Authors:** Harriet A. Ball, James Stevens, Julian D. Gillmore

**Affiliations:** 1 North Bristol NHS Trust, Southmead Hospital, Southmead Road, Westbury-On-Trym, Bristol, BS10 5NB UK; 2 Bristol Medical School, University of Bristol, Bristol, UK; 3 National Amyloidosis Centre, University College London, London, UK

**Keywords:** Peripheral neuropathy, Amyloidosis, Transthyretin, Domino liver transplant

## Abstract

**Background:**

Peripheral neuropathy caused by amyloidosis is one of the well-recognised sequelae of mutations in the transthyretin gene (*TTR*).

**Case presentation:**

We describe a case of peripheral neuropathy in a White British 74 year old man with wild-type *TTR*, 8 years following receipt of a ‘domino’ liver transplant (from a donor with a *TTR* mutation). The clinical phenotype and neurophysiology, coupled with presence of ATTR amyloid deposits on fat biopsy, established the diagnosis of ATTR amyloid neuropathy, as a consequence of receipt of a variant-TTR secreting liver. A nerve biopsy was not clinically appropriate for this patient. Such cases are rare since recipients of such livers are typically restricted to people whose natural lifespan is unlikely to stretch into the anticipated symptomatic period of ATTR amyloidosis. However, novel “gene silencing” therapeutics are now available which can dramatically alter the course of this disorder, by reducing the proportion of abnormal proteins.

**Conclusions:**

This represents a rare but predictable iatrogenic side effect, and doctors should be aware of this eventuality occurring in a shorter time span than previously anticipated.

## Background

Hereditary ATTR amyloid polyneuropathy (hATTR-PN) is a rare inherited condition. Mutations in the *TTR* gene cause the liver to produce abnormal TTR protein, which accumulates as deposits in body tissues (amyloidosis) which can cause tissue damage. Peripheral neuropathy, autonomic neuropathy, and cardiomyopathy are common consequences of amyloid deposition [1]. Because transthyretin is mainly produced in the liver, liver transplant has been a management option, since it reduces the production of additional amyloid deposits, if undertaken early in the condition.

**Fig. 1:**
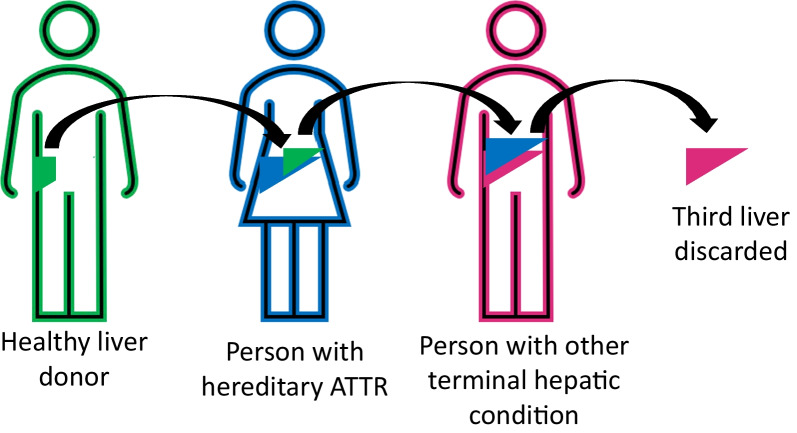
HYPERLINK "sps:id::fig1||locator::gr1||MediaObject::0" “Domino” liver transplant. Our case is represented as the final person in the sequence, he received a liver that is functioning well, but is slowly producing variant transthyretin, eventually leading to amyloidosis

Due to a severe shortage of graft tissue, it has been common for hATTR patients who receive a donor liver, to pass on their histologically-normal but variant-TTR-producing liver, to a patient who needs a transplant for a separate reason (Fig. 1). By the end of 2019, at least 2294 such “domino” liver transplants had occurred [2].

Patients born with the *TTR* mutation do not develop symptoms for at least 20 years [3]. Recipients of variant-TTR livers are generally chosen with a lifespan not expected to be adversely affected, with this timespan in mind. However, over recent years, TTR neuropathy has been noted in a subset (up to one-sixth) [4] of donor recipients after a considerably shorter interval. The aim of this report is to describe a case for whom peripheral neuropathy due to variant-TTR acquired from a “domino” donated liver, progressed much faster than in those who inherit a *TTR* mutation, and caused significant disability.

## Case presentation

The White British patient received a domino liver transplant for non-alcoholic steatohepatitis when he was 66 years old (in 2009). The donor of the liver had the most common form of the *TTR* mutation (Val30Met). The recipient was a never-smoker and had no significant alcohol history. He had a history of type 2 diabetes mellitus (diagnosed at age 62, with retinopathy from age 66) and hypertension. He subsequently developed chronic kidney disease with exacerbations, low vitamin B12 replaced with injections, pulmonary embolism, paroxysmal supra-ventricular tachycardia, heart failure, transient ischaemic attack, and episodes of weight loss. At the point of referral to outpatient neurology (age 76), he was taking tacrolimus and prednisolone for the transplant, plus medications to manage his vascular risk, clotting tendency, diabetes and heart failure, amongst other problems (see Table 1). He was widowed, and a retired office worker. He lived alone with carer support 5 times per week.

**Table 1:** Medication list at time of referral to neurology

Medication	Oral dose (daily unless specified otherwise)
Tacrolimus	1 mg (twice daily)
Prednisolone	5 mg
Rivaroxaban	20 mg
Fludrocortisone	200 mcg
Eplerenone	25 mg
Digoxin	125 mcg
Atorvastatin	40 mg
Nebivolol	10 mg
Linagliptin	5 mg
Esomeprazole	40 mg
Finasteride	5 mg
Tamsulosin	400 mcg
Multivitamin Sanatogen A-Z	1

Medications taken less frequently	
B12 injections, codeine, paracetamol, topical gels for legs	

He first noticed neuropathic symptoms 8 years post-transplant (at age 74), alongside some weight loss. This began as a numbness and paraesthesia in his feet, which progressed over 2 years to include sensory involvement of his knees then hands, and motor involvement in his ankles. His mobility became significantly impaired, at some times needing a wheelchair; this was complicated by chronic infection of the right knee and calf, and development of a Charcot knee joint. He had also noticed some dizziness on standing, but without loss of consciousness; he had not experienced gastrointestinal disturbance.

On examination, he had blood pressure 143/78 and heart rate 68, with no significant postural blood pressure drop. He had a moderate to severe peripheral neuropathy, including bilateral reduced ankle dorsiflexion and plantar flexion (power MRC grade 1/5) as well as reduced inversion and eversion. In the upper limbs, power was intact aside from MRC grade 4/5 of first dorsal interosseous and adductor digiti mimimi bilaterally. Pinprick and light touch were impaired in a length-dependent manner as far as his knees and over his fingers. Vibration sense was impaired as far as the anterior superior iliac spines.

He had evidence of impaired renal function [serum creatinine (130 µmol/L), low creatinine clearance (31 mL/min), raised albumin:creatinine ratio (56 mg/mmol), and urinary protein loss (0.6 g/day)]. Immunoglobulins were normal, with no paraprotein. Bence Jones protein was absent and serum free light chain kappa:lambda ratio was normal. N-terminal pro-Brain Natriuretic Peptide (NT-proBNP) was raised at 938 ng/L. He had normal liver function tests.

Nerve conduction studies were not possible in the lower limbs due to significant oedema and chronic skin changes. In the upper limbs, superficial sensory responses were unobtainable; motor responses in general showed mild slowing (median nerve velocity 35 m/s) but more pronounced loss of amplitude (median nerve 0.5 mV). Overall, this was considered suggestive of a predominantly axonal polyneuropathy.

Peripheral nerve biopsy was not undertaken due to concerns over poor healing. In the context of this and the ATTR status of his donor liver, he was referred to the UK National Amyloidosis Centre. Serum Amyloid P component (SAP) scintigraphy showed no evidence of visceral amyloid deposits. There was no evidence of cardiac amyloidosis on echocardiography or diphosphono-1,2-propanodicarboxylic acid (DPD) scintigraphy. A fat aspirate contained ATTR amyloid deposits, typed by mass spectrometry. Minimally invasive biopsies are usually preferred over nerve biopsies for the diagnosis of ATTR amyloid neuropathy [5]; in this case the diagnosis was highly likely given the identification of amyloid deposits alongside the clinical presentation.

Regarding potential alternative explanations for his peripheral neuropathy, his vitamin B12 level had been well controlled by injections. He had suffered from diabetes since 4 years prior to his liver transplant, and only experienced minor neuropathic symptoms 8 years after the transplant, which then rapidly progressed over the next 2 years. His glycated haemoglobin (HbA1C) level was 49 mmol/mol and had been at a similar level throughout. As such, diabetes was considered unlikely to be the sole cause of such a rapidly progressive and profound sensori-motor neuropathy.

Initial management was supportive. In light of the confirmation of presence of ATTR amyloid deposits and the classical clinical phenotype, the patient was commenced on patisiran, a small interfering RNA therapeutic, at the standard dose of 0.3 mg/kg every 3 weeks. However, he then suffered a stroke resulting in impaired mobility and necessitating cessation of patisiran treatment. The peripheral neuropathy had remained stable on examination for the duration of patisiran treatment, and it was too brief to expect clinical improvement. Four years after the onset of neuropathic symptoms, he passed away secondary to complications of his comorbidities (including the stroke).

## Discussion and conclusions

We describe an older gentleman with a sensory-motor axonal length-dependent polyneuropathy, which progressed too far and too aggressively over 2 years to be easily explicable by age or well-controlled diabetes. He had been the recipient (10 years previously) of a liver from a donor who had familial amyloidosis. The presence of proven ATTR amyloid deposition elsewhere in the recipient is consistent with the most likely explanation of the presentation—as ATTR amyloid neuropathy.

Although domino liver transplantation is now rare, this case gives insights into the pathophysiology of amyloid neuropathy. Explanations for the variability in disease latency may include age [6], existence of fibrils in the transplanted liver [7], and/or the effects of surgery, inflammatory reactions or immunosuppressant drugs [3, 8]. Our case is particularly relevant in highlighting that complications of hATTR domino liver transplant can occur in considerably fewer years than the typical age at which such complications occur in those people who inherit this genetic liability.

The commonest inherited form of ATTR amyloidosis (Val30Met) affects the peripheral and autonomic nerves and often spares the heart, particularly in patients whose disease-onset occurs before 50 years of age [9]; the same appears to be true in a case series of recipients of donor livers from patients with neuropathic Val30Met amyloidosis [4], though long-term follow up data is limited.

Diagnosis of polyneuropathy due to ATTR can include *TTR* gene sequencing in those who inherit the variant, and tools to confirm amyloid deposits: biopsy, and bone scintigraphy with DPD, hydroxymethylene diphosphonate, or pyrophosphate [5]. Finding amyloid deposits can be difficult, and minimally invasive biopsy (such as salivary gland, skin, or abdominal fat tissue) can be favoured over nerve or cardiac biopsy.

Recent research has shown the utility of patisiran to reduce both variant and wild-type TTR in the plasma of patients with hereditary ATTR amyloidosis [10]; and subsequently a phase III study showed improvement in indices of neuropathy and quality of life in such patients [11]. In the UK, the National Institute for Health and Care Excellence (NICE) has recommended the use of this medication as an option for managing this condition, despite its high price, due to considerations including the rarity and severity of the condition, its large effect on patients and care-givers, the size of the health benefits of the treatment (not all of which are easily measured) and the innovative nature of the treatment [12]. The existence of such therapies implies a need for careful clinical review of patients who may be at risk of ATTR amyloidosis.

This case demonstrates that careful phenotyping of a peripheral neuropathy, and its time-course, can help narrow the differential diagnosis. In recipients of donor organs with a new clinical problem, we should consider not only the effects of compatibility and immunosuppression, but also, potential disorders (infective or genetic) that may have been transmitted alongside the donated organ.

## Data Availability

Data sharing is not applicable to this article as no datasets were generated or analysed during the current study.

## Abbreviations

*TTR/*TTR: Transthyretin *gene*/protein
ATTR: Amyloidogenic transthyretin
hATTR-PN: Hereditary amyloid polyneuropathy
MRC: Medical Research Council power grade
DPD: Diphosphono-1,2-propanodicarboxylic acid
